# Effects of Variable Embryo Transfer on the Variable Window of Implantation and Analysis of Pregnancy Outcomes

**DOI:** 10.7759/cureus.60020

**Published:** 2024-05-10

**Authors:** Deepti Shrivastava, Shilpa Dutta

**Affiliations:** 1 Obstetrics and Gynaecology, Jawaharlal Nehru Medical College, Datta Meghe Institute of Higher Education and Research, Wardha, IND; 2 Clinical Embryology, Jawaharlal Nehru Medical College, Datta Meghe Institute of Higher Education and Research, Wardha, IND

**Keywords:** implantation, ivf, rif, window of implantation, infertility, embryo transfer

## Abstract

Background

A descriptive analysis of patients who underwent various embryo transfer methods to address the root cause of their infertility at a tertiary infertility care complex in Wardha, India, is presented herein. This analysis aims to evaluate the management of infertility and assess pregnancy outcomes.

Methodology

We conducted a retrospective cohort study on patients who underwent various embryo transfer methods to address the cause of their infertility, specifically focusing on a variable window of implantation (WOI) at a tertiary infertility clinic over a one-year period. The medical records of 11 patients in both the variable embryo transfer (VET) and control groups were reviewed and analyzed for this article.

Results

The examination of medical records revealed a significant improvement in the rate of implantation (p-value = 0.04) and clinical pregnancy outcomes (p-value = 0.03) among patients who underwent VET. Comparable statistical outcomes were observed for other variables of pregnancy outcome, including miscarriage rate, multiple pregnancy rate, and biochemical pregnancy rate.

Conclusion

This retrospective cohort study suggests that the utilization of VET could be a viable option for women experiencing recurrent implantation failure cycles, particularly when an adequate number of embryos are available. This is owing to the challenges in clinically diagnosing a variable WOI. Further studies with a significantly larger sample population are recommended to validate the results and integrate this approach into the standard operating procedures, aiming to enhance the likelihood of pregnancy in these populations.

## Introduction

It has been reportedly found that around 8 to 12% of the reproductive age group of couples are suffering from infertility around the world [1,2]. One of the critical factors responsible for causing infertility is implantation failure. During the natural process of the menstrual cycle, the endometrial lining of the uterus is prepared both structurally and functionally to receive the embryo for successful conception [3]. Implantation is basically the dialogue shared between the embryo and the endometrial wall. It is a requisite step to initiate the apposition, adhesion, and invasion of the blastocyst into the endometrial wall of the uterus [3,4]. Major artificial reproductive techniques have been found to focus on the identification of the competent embryo that needs to be transferred for the purpose of implantation. However, the focus has been less on the other end of the story, the endometrium [3]. Although we have a few protocols for the identification of uterine receptivity, such as preimplantation genetic testing for aneuploidy (PGT-A) and time-lapse technology, they’re not routinely assessed in the majority of the centers [3].

It has been observed that around days 19 to 21 of the typical 28-day menstrual cycle, the endometrium is more receptive toward the reception of the embryo than the other phases [3,5]. This receptivity is brought on by the effect of master hormones such as estrogen and progesterone [6,7]. For successful implantation, it is crucial for the embryo and window of implantation (WOI) to be synchronized. Hence, to have fruitful in vitro fertilization (IVF) results, it is essential to track the WOI in patients [3]. The endometrial receptivity analysis test (ERA test) has been found in the literature to distinguish receptivity into three categories such as pre-receptive, receptive, and post-receptive [8]. However, the result of this test fails to provide fruitful results in the case of patients with a variable WOI. In the population, the majority of the females present with asynchronous menstrual cycles, which is attributed to having a variable WOI. Hence, the result of the ERA test sometimes fails to provide the correct window for the next menstrual cycle. This results in implantation failure in case of embryo transfer on day 6 of progesterone as per routine protocol, as the endometrium might be in the pre-receptive phase of the post-receptive phase [8].

Hence, in order to tackle the enigma of the variable WOI, this study has been conducted to analyze the efficacy of variable embryo transfer (VET) on the WOI and, thereby, analysis of the pregnancy outcome as a result of the procedure. Our study is probably the first among the existing literature to conduct VET on a significant sub-fertile population who have suffered from infertility due to variable WOI.

## Materials and methods

This retrospective cohort research study consisted of being carried out over a span of a year at a tertiary infertility care complex in Wardha, India. The study included a total of 11 patients who underwent VET and another set of 11 patients who underwent conventional embryo transfer, serving as the control group. Data for all eligible patients were gathered from hospital records, including laboratory test reports, doctor’s consultation reports, ultrasonography scans, hysteroscopy reports, ERA test reports, and the patient's previous medical history. Patients who lost follow-up during the treatment were excluded from the study. The medical records of patients were screened for demographic profiles.

Ethical consideration

This study has been conducted after taking due ethical clearance from the Institutional Ethical Committee of Datta Meghe Institute of Higher Education and Research, Wardha, India, having IEC no. DMIHER(DU)/IEC/2023/1320, dated September 8, 2023.

Inclusion criteria

Patients undergoing infertility treatment at the tertiary infertility care complex in Wardha, India, who underwent either VET or conventional embryo transfer. Availability of complete medical records including laboratory test reports, doctor’s consultation reports, ultrasonography scans, hysteroscopy reports, ERA test reports, and previous medical history. Patients with complete demographic profiles are documented in their medical records.

Exclusion criteria

Patients who lost follow-up during the treatment process, as their data may be incomplete or unreliable for analysis. Patients with incomplete medical records, lack essential information such as laboratory results or previous medical history, making it difficult to assess their infertility treatment outcomes accurately.

Statistical analysis

IBM SPSS Statistics for Windows, Version 26 (Released 2019; IBM Corp., Armonk, New York, United States) served as the software for every statistical computation. A two-tailed t-test was the method of choice in order to evaluate the demographic data, whilst a z-test was implemented to analyze the pregnancy results. Similarly, p < 0.05 has been set as the statistical significance criterion.

Methodology

Controlled Ovarian Stimulation and Ovum Aspiration

Upon reviewing the medical records, we found that all patients underwent stimulation using the standard gonadotrophin-releasing hormone (GnRH) long protocol (3.75 mg) along with recombinant follicle-stimulating hormone (rFSH) (150-300 U/day), until they observed at least three or more mature follicles measuring around 18 mm. Triggering was achieved using a subcutaneous injection of human chorionic gonadotrophin (hCG) of 10,000 IU, and the ovum was aspirated 36 hours after the trigger.

Intracytoplasmic Sperm Injection (ICSI) Followed by Embryo Culture

Denudation of retrieved oocytes occurred, and ICSI was performed two to three hours post-retrieval from the follicular fluid. The readily apparent clear emergence of pronuclei and the second polar body, 16-18 hours following the ICSI technique, implied fertilization. In a benchtop incubator, the embryos were fostered utilizing a one-step culture medium.

Embryo Grading and Vitrification

Embryos exhibiting an even size of six to eight cells with no or less than 10% fragmentation, along with no multinucleation, were termed Grade I cleavage-stage embryos. Embryos with a tightly packed trophectoderm and a crescent-shaped inner cell mass at day 5 were graded as blastocysts. They were vitrified using a globally acknowledged vitrification kit.

Embryo Transfer and Luteal Support

All embryo transfers took place in the patients' natural cycles. Patients who underwent VET received day 3 and day 5 embryos on day 6 of progesterone support, while the control group received two day 5 embryos on day 6 of progesterone.

Clinical pregnancy was confirmed using the β-hCG test 14 days after embryo transfer. Intrauterine pregnancy was validated by transvaginal ultrasonography conducted 10 days later. The presence of a fetal heart rate during the six to eight weeks of the gestational period confirmed clinical pregnancies.

## Results

Table 1 outlines the demographic characteristics of patients who underwent VET and conventional transfer. The data were collated from the medical records. Statistical analysis using p-values found no substantial differences among the variables, which include age, basal metabolic index (BMI), FSH, duration of infertility, anti-Müllerian hormone (AMH) value, primary infertility, secondary infertility, abortion rate, previous failed cycles, sperm count, and motility.

**Table 1:** Baseline characteristics of the patients A p-value <0.05 is considered statistically significant and the statistical analysis was conducted using an independent samples t-test FSH: follicle-stimulating hormone; AMH: anti-Müllerian hormone; IVF: in vitro fertilization; VET: variable embryo transfer

Variables	VET	Controlled group	p-value
No. of patients	11	11	
Age (years)	36.8±2.6	35.5±3.1	0.398
Body mass index (BMI) (kg/m^2^)	25.71±0.21	26.03±0.36	0.125
Basal FSH level day 3 (IU/L)	6.1±2.1	6.4±0.16	0.663
Duration of infertility (years)	7.14±1.3	7.58±1.5	0.485
AMH (ng/mL)	3.6±1.12	3.71±1.23	0.826
Primary infertility (%)	63.6 (7/11)	72.7 (8/11)	0.4872
Secondary infertility (%)	36.4 (4/11)	27.3 (3/11)	0.967
Abortion (%)	36.4 (4/11)	36.4 (4/11)	0.988
Previous failed IVF cycles	2.23±0.3	2.36±0.61	0.319
Sperm count (M/mL)	51.48±2.5	53.34±2.3	0.072
Sperm motility (%)	41.54±1.781	41.43±1.691	0.906

Table 2 illustrates the characteristic parameters of ovarian controlled stimulation along with ICSI results between the VET and control groups, along with p-values indicating statistical differences. Top-quality blastocyst, endometrial thickness on embryo transfer day, fertilization rate, number of oocytes, number of metaphase II (MII) oocytes, endometrial thickness, and gonadotropin dosage constitute some of the variables subsequently measured. Although notable numerical differences were observed among the compared groups, the p-values suggest that these numerical differences were not statistically significant.

**Table 2:** Characteristics of ovarian stimulation and ICSI result A p-value <0.05 is considered significant and the Mann-Whitney U-test was utilized for statistical analysis VET: variable embryo transfer; ET: embryo transfer; MII: metaphase II; ICSI: intracytoplasmic sperm injection

Variables	VET	Controlled group	p-value
Gonadotropin dosage (IU)	2753±103.2	2872±98.6	0.221
Number of oocytes	10.65±0.624	10.89±0.718	0.479
Number of MII oocyte	8.205±0.89	8.365±1.02	0.705
Fertilization rate (%)	62.7±14.3	60.2±16.8	0.71
Day 3 embryo (%)	72.35±3.02	73.55±2.84	0.348
Top-quality blastocyst (%)	63.71±2.87	65.11±2.40	0.711
Endometrial thickness on ET day (mm)	8.920±1.4	9.20±1.6	0.662

Table 3 represents the comparative analysis of the pregnancy outcomes among the two groups. It included implantation rate, biochemical pregnancy rate, clinical pregnancy rate, multiple pregnancy rate, and miscarriage rate. The VET group (54.5%, 6/11) has a significantly higher implantation rate than the control group (36.4%, 4/11). However, the biochemical pregnancy rate did not exhibit any statistical difference between the two groups: VET (45.45%, 5/11) and the control group (27.27%, 3/11), with a p-value of 0.53. The clinical pregnancy rate in the VET group has been observed as much higher (54.5%, 6/11) than that of patients in the controlled group (18.18%, 2/11), with a p-value of 0.03. The VET group has a multiple pregnancy rate of 18.18% (2/11) compared to the controlled group with 9.09% (1/11), but the difference is not significant statistically (p-value = 0.42). Lastly, the miscarriage rate in VET is 9.09% (1/11), while for the controlled group, it has been observed much higher at 18.18% (2/11), but p-value = 0.18 signifies that this difference does not reach statistical significance levels. These findings suggest that the VET group presents advantageous results concerning implantation and clinical pregnancy rates with similar percentages of biochemical pregnancies, multiple pregnancies, and miscarriages compared to the controlled group.

**Table 3:** Pregnancy outcomes among the compared groups A p-value <0.05 is considered significant and the chi-square test was used for statistical analysis VET: variable embryo transfer

Variable	VET	Controlled group	p-value
Implantation rate (%)	54.5% (6/11)	36.4% (4/11)	0.04
Biochemical pregnancy rate (%)	45.45% (5/11)	27.27% (3/11)	0.53
Clinical pregnancy rate (%)	54.5% (6/11)	18.18% (2/11)	0.03
Multiple pregnancy rate (%)	18.18% (2/11)	9.09% (1/11)	0.42
Miscarriage rate (%)	9.09% (1/11)	18.18% (2/11)	0.18

## Discussion

Stamenov et al. were the pioneer researchers who reportedly made the first move with the implementation of a mixed double embryo transfer method on a patient having a variable WOI [9]. They reported on a case of a 37-year-old woman suffering from recurrent implantation failure (RIF), previously reported to have an idiopathic cause of infertility and later diagnosed with the variable WOI, which was confirmed by endometrial biopsy on subsequent three menstrual cycles. They simultaneously transferred day 3 and day 5 embryos on day 7 post-ovulation. The woman was reportedly pregnant with a single embryo as validated by ultrasound, and thereafter, she delivered a healthy baby girl [9]. This case provided a unique avenue to perform further investigation on a significant population. It has been observed that more than 85% of IVF failure cases have been attributed to implantation failure [10]. This has been reported as caused due to having a variable WOI in the majority of the patients. Upon analyzing the available literature, we have found that mainly embryo transfers have failed to culminate into successful implantation as on day 6 of embryo transfer as per protocol, the endometrium may have been in either a pre-receptive state or post-receptive state due to which the blastocyst that we transfer degenerates. There are discrepancies present in the existing literature regarding the ways of tackling the glaring condition, which has been reportedly providing hindrance to the success rate of IVF treatment.

WOI refers to the period during the menstrual cycle in which the endometrium remains highly receptive to the embryo in order to culminate into implantation [11,12]. Several scientists have pointed toward having a synchronous cross-talk between the embryonic and endometrial layers as crucial for establishing embryo implantation on the uterine wall. However, the detailed molecular mechanism that might have been present to result in this phenomenon is yet to be confirmed by the scientific world. A review article formulated by Sun and Yeh states that there might be a five-step mechanism that may lead to the formation of the WOI. They are appropriate synchronicity between endometrial cells, acceptable crosstalks between the endometrial layer and the embryo, standard progesterone signaling and endometrial responses to serum progesterone, silent genetic variants, and typical physical morphology of the endometrial glands [11].

Ideally, the WOI opens around six to seven days post-ovulation period [13,14]. However, due to a significant number of asynchronous menstrual periods, women are on the rise nowadays; hence, the ideal WOI remains questionable in those cases, which leads to RIF. Several studies have been conducted to overcome the enigma of an erratic WOI. A recent approach known as sequential embryo transfer is based on the module that increases the time period of hitting the WOI. However, several studies have reported that there are no significant differences observed for RIF patients. This study has a hypothesis that transferring day 3 (cleavage) and day 5 (blastocyst) on day 6 of progesterone has maximum chances of pregnancy as many studies have reported that patients with a variable WOI fail to conceive because majorly the endometrium became pre-receptive stage during the conventional period of embryo transfer [15,16]. Transferring the day 3 embryo ensures that if the endometrium is in the pre-receptive stage during the conventional day of embryo transfer, then once it grows into the day 5 embryo in vivo, the endometrium will be ready to receive the embryo for implantation. Therefore, VET serves as an insured method to at least get one of the embryos implanted in patients suffering from RIF. However, it is also important to note that there is a potential chance of having multiple pregnancies in the case of VET as if the endometrium is receptive during the conventional period, there are certain chances that both the embryos may get implanted due to embryo-uterine crosstalk.

Limitations

This study, conducted at a tertiary infertility center, provides thought-provoking new insights into the world of infertility research, but it also has some limitations to consider. Firstly, its retrospective cohort design means that it relies on past medical records, which may have inconsistencies or missing information. Additionally, the study's sample size is small, comprising only 11 patients, which may not accurately reflect the diversity of cases encountered in larger populations. Moreover, being a single-center study, its findings may not be applicable universally and could be influenced by specific center practices or patient demographics. These limitations suggest caution in interpreting the results, highlighting the need for further research with larger, more diverse samples to confirm the efficacy and generalizability of VET methods in addressing RIF.

## Conclusions

In summary, this retrospective cohort study suggests that the VET method could be a promising approach in the field of assisted reproductive technology (ART) to address the challenge of an erratic WOI in women with a sufficient number of embryos. This may contribute to improving the chances of a successful pregnancy outcome for patients experiencing implantation failure due to a variable WOI. We recommend further validation of these findings by conducting studies on a larger sample population, such as randomized controlled trials (RCTs), to establish this procedure as a standard protocol in ART for scenarios involving variable WOIs.
